# Myoferlin contributes to invasiveness of human T-cell leukemia virus type 1-infected T cells

**DOI:** 10.1128/jvi.00264-26

**Published:** 2026-04-21

**Authors:** Md Abu Kawsar Sarker, Nicholas Polakowski, Camila Sagasti, Kimson Hoang, Gabriel Hugh Gibson, Isabelle Lemasson

**Affiliations:** 1 Department of Microbiology and Immunology, Brody School of Medicine, East Carolina University 3627, Greenville, North Carolina, USA; 2 Laboratorio de Inmunovirologia, Institut Pasteur de Montevideo 123939, Montevideo, Uruguay; 3 Unidad Académica de Inmunobiología, Facultad de Medicina, Universidad de la República 54795 , Montevideo, Uruguay; Icahn School of Medicine at Mount Sinai, New York, New York, USA

**Keywords:** myoferlin, migration, leukemia, HTLV-1, COTL1

## Abstract

The retrovirus human T-cell leukemia virus type 1 (HTLV-1) predominantly infects CD4^+^ T cells *in vivo*. Two of the primary diseases caused by HTLV-1 infection are an aggressive leukemia designated adult T-cell leukemia (ATL) and a progressive neurodegenerative disease known as HTLV-1 associated myelopathy/tropical spastic paraparesis (HAM/TSP). For both diseases, an enhanced infiltration capacity of the HTLV-1^+^ T cells contributes to pathogenesis. This phenotype arises from changes in gene expression elicited by viral proteins. We recently reported that the viral protein, HBZ, activates expression of myoferlin (MyoF), which is overexpressed in several epithelial cancers where it promotes tumor cell metastasis. To test whether MyoF also contributes to the infiltration capacity of HTLV-1^+^ T cells, we performed *in vitro* adhesion, migration, and invasion assays using HTLV-1-transformed T cells. MyoF knockdown or inhibition in these cells reduced their adhesion to an endothelial cell monolayer and similarly diminished their invasion properties. RNA-seq analysis showed that knockdown of MyoF in HTLV-1-infected cells reduced expression of several genes involved in these processes, among which was COTL1 (coactosin-like protein 1). COTL1 binds and stabilizes filamentous actin and has been reported to both promote and inhibit tumor cell metastasis. In HTLV-1-infected cells, the effect of knocking down COTL1 was restricted to reducing cell invasion through a reconstituted basement membrane. From these results, we propose that MyoF contributes to the infiltration capacity of HTLV-1^+^ T cells, in part, by affecting gene expression, with MyoF-enforced expression of COTL1 specifically facilitating HTLV-1^+^ T-cell migration through extracellular matrices.

**IMPORTANCE:**

CD4^+^ T cells infected with human T-cell leukemia virus type 1 (HTLV-1) display an enhanced ability to infiltrate into tissues and organs, sometimes leading to pathological effects in patients infected with the virus. We provide evidence that abnormal expression of the cellular protein myoferlin (MyoF) in HTLV-1-infected T cells contributes to this phenotype, in part, by directing changes in the expression of other cellular genes. One gene on which MyoF produces a positive effect on expression was found to be COTL1, which encodes the F-actin-binding protein, coactosin-like protein 1. Our results indicate that COTL1 plays a specific role in the migration of HTLV-1-infected T cells through extracellular matrices, which is a critical step in the process of tissue infiltration.

## INTRODUCTION

There is estimated to be 5–10 million people worldwide who are infected with the retrovirus human T-cell leukemia virus type 1 (HTLV-1) (1). *In vivo*, HTLV-1 primarily infects CD4^+^ T cells (2), and while most carriers remain asymptomatic during their lifetime, a small percentage experience pathological effects associated with the viral infection. HTLV-1-associated disorders present as inflammatory or pulmonary diseases or leukemia (3, 4). Two of the primary diseases directly caused by the viral infection are HTLV-1-associated myelopathy or tropical spastic paraparesis (HAM/TSP) and adult T-cell leukemia/lymphoma (ATL), which develop in approximately 5% of carriers (5–8). HAM/TSP is a progressive neurodegenerative disorder caused by demyelination of axons in the central nervous system. In contrast, ATL is a fatal malignancy characterized by an uncontrolled proliferation of HTLV-1^+^ CD4^+^ T cells.

The HTLV-1-encoded protein, HBZ, is believed to contribute to the development of ATL and subsequent maintenance of the leukemic phenotype (9, 10). In addition, HBZ enhances cell contact-dependent infection of HTLV-1 (11, 12). These effects are not directly mediated by HBZ but rather result from the functions of cellular genes that are activated by the viral protein. Indeed, through localization to specific chromatin sites and interactions with a variety of cellular transcriptional regulators, HBZ regulates cellular gene transcription (13, 14). In the context of viral infection, HBZ was recently found to activate transcription of the *MYOF* gene, which encodes myoferlin (MyoF), a member of the Ferlin family (12). Each Ferlin is comprised of a C-terminal transmembrane domain that anchors the protein to intracellular membranes and a large N-terminal cytoplasmic-exposed region that contains multiple C2 domains and plays a general role in membrane repair (15). In addition to membrane repair, MyoF has been found to regulate such processes as endosomal trafficking and mitochondrial dynamics (16).

MyoF overexpression in several epithelial cancers is associated with a poor patient prognosis (16–22). In these cancer cells, MyoF enforces effective growth factor signaling to stimulate cell proliferation and activate the epithelial to mesenchymal transition (EMT) (21, 23–27). This latter process leads to metastasis, allowing cancer cells to detach from the solid tumor and invade other host tissues. Interestingly, an invasive capacity also embodies HTLV-1^+^ T cells associated with both HAM/TSP and ATL. For HAM/TSP, infected T cells exhibit a high migratory activity, which is believed to allow them to traverse the blood-brain barrier and initiate an inflammatory response in the central nervous system (28). For ATL, the leukemic cells are known to infiltrate the skin, bone, and visceral organs, where they cause lesions and other adverse effects (29). The initial stages related to the infiltration properties of HTLV-1^+^ T cells are believed to involve overactivation of the normal extravasation migration processes of T cells during an immune response (28, 30).

Based on these observations, in this study, we analyzed whether abnormal expression of MyoF contributes to the cell adhesion, migration, and invasive properties of HTLV-1-transformed T cells. First, we found that knockdown of MyoF in HTLV-1-transformed T cells reduced cell adhesion to an endothelial monolayer. Similar effects were observed using a specific inhibitor of MyoF, WJ460. Using *in vitro* assays, we also found that knockdown of MyoF reduced the ability of cells to cross a reconstituted basement membrane and, separately, an endothelial monolayer, which is indicative of a reduction in invasion. Through RNA-seq analysis, we identified genes involved in cell adhesion, actin dynamics, and/or cell migration that showed a reduction in RNA levels when MyoF was knocked down. For a subset of these genes that were also relevant to ATL (*BCL2*, *COTL1*, *ITGA4*, *S100A4*, *SCD4,* and *STAT3*), we confirmed that levels of the corresponding proteins were also reduced in MyoF knockdown cells. We then focused on *COTL1*, which encodes coactosin-like F-actin binding protein 1, a protein that stabilizes filamentous actin (F-actin). As expected, knockdown of COTL1 in HTLV-1-transformed T cells reduced the levels of F-actin, which was similarly observed with knockdown of MyoF. However, unlike with MyoF knockdown, knockdown of COTL1 did not affect invasion through an endothelial monolayer and actually increased cell adhesion to the monolayer. In contrast, knockdown of COTL1 reduced invasion through a reconstituted basement membrane. Based on these results, we propose that COTL1 contributes to actin remodeling required for HTLV-1-transformed T cells to navigate through extracellular matrices. This function may contribute to the pathological effects of HTLV-1 infection associated with the enhanced infiltration capabilities of HTLV-1^+^ T cells.

## RESULTS

### Knockdown of MyoF reduces HTLV-1 T-cell adhesion to endothelial cells

**Fig 1:**
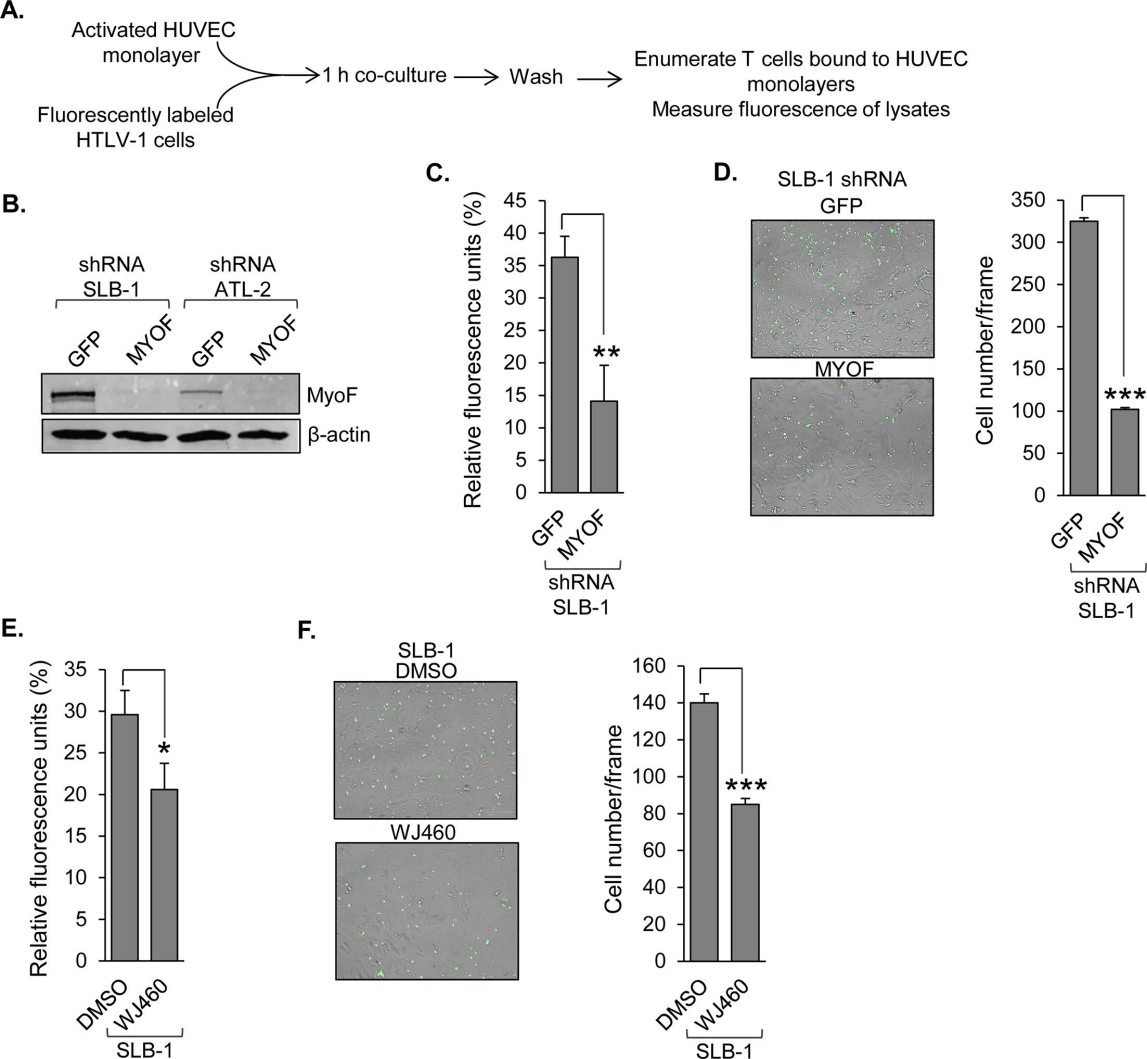
MyoF depletion reduces HTLV-1-infected SLB-1 T-cell adhesion to HUVEC monolayers. (**A**) The flow diagram shows assays used to test cell adhesion. Calcein AM-labeled HTLV-1-transformed T cells stably expressing an shRNA targeting GFP (negative control) or MYOF mRNA were cultured on HUVEC monolayers and lysed for fluorescence analysis or visualized by microscopy. Separately, T cells were treated with DMSO or 1 μM WJ460 (a MyoF inhibitor). (**B**) MyoF expression in control (GFP) and MyoF knockdown SLB-1 and ATL-2 cells. Whole cell extracts (40 μg) were analyzed by western blot using antibodies against MyoF and β-actin. (**C**) The graph shows fluorescence values of co-culture lysates from assays using shRNA-expressing SLB-1 cells. Values are averaged from three replicates and are representative of three independent experiments. (**D**) A representative image of Calcein AM-labeled, shRNA-expressing SLB-1 cells bound to HUVEC monolayers (bright field), with the number of bound T cells shown in the graph. (**E**) The graph shows fluorescence values of co-culture lysates from assays using DMSO- or WJ460-treated SLB-1 cells. (**F**) A representative image of Calcein AM-labeled, DMSO- or WJ460-treated SLB-1 cells bound to HUVEC monolayers (bright field), with the number of bound T cells shown in the graph. Graphs in panels C and E show values averaged from three replicates and are representative of three independent experiments. Graphs in panels D and F show the number of cells averaged from three separate fields for each adhesion condition. * *P* < 0.05, **P < 0.01, ***P < 0.001 (*t*-test).

**Fig 2:**
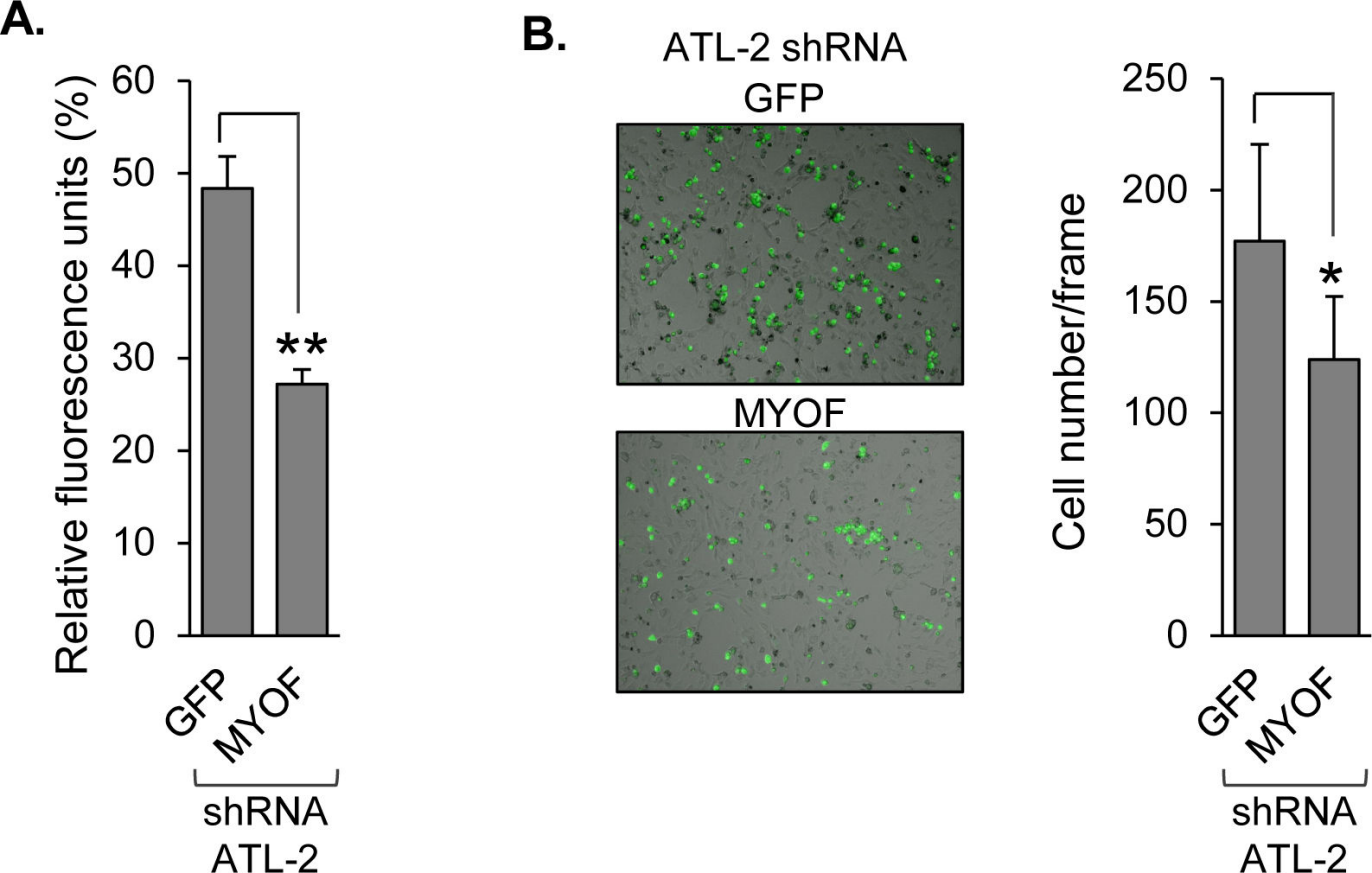
MyoF depletion reduces HTLV-1-infected ATL-2 T-cell adhesion to HUVEC monolayers. (**A**) The graph shows fluorescence values of co-culture lysates from assays using shRNA-expressing ATL-2 cells averaged from three replicates and is representative of two independent experiments; ** *P* < 0.01 (*t*-test). (**B**) A representative image of Calcein AM-labeled, shRNA-expressing ATL-2 cells bound to HUVEC monolayers (bright field). The graph shows the number of cells averaged from eight fields for each knockdown. * *P* < 0.05 (Mann-Whitney U test).

In the context of cell contact-dependent HTLV-1 infection, we previously reported that knockdown or inhibition of MyoF in HTLV-1-infected T cells reduces adhesion to CHO cells (12). Surprisingly, though, the central cell surface-binding partners involved in viral infection, ICAM-1 and LFA-1 (31), were not responsible for this effect. Based on these observations, we pursued the role of MyoF in HTLV-1-infected T-cell adhesion using human umbilical vein endothelial cells (HUVECs), with the aim of using an *in vitro* model that better relates to the tissue infiltration properties of HTLV-1^+^ T cells than CHO cells. To analyze the effect of MyoF on adhesion to HUVECs, we compared HTLV-1-infected cells, SLB-1, stably expressing an shRNA targeting the *MYOF* gene transcript (shMYOF), to control SLB-1 cells stably expressing an shRNA targeting GFP (shGFP; Fig. 1B) (12). These cells were labeled with Calcein AM and added to activated HUVEC monolayers, which were then washed to remove unbound T cells. Monolayer lysates or intact monolayers were tested for fluorescence signal or bound T cells, respectively (Fig. 1A). Prior to these experiments, we determined that there is no difference in Calcein AM staining between shGFP and shMYOF cells (Fig. S1A). Knockdown of MyoF reduced cell adhesion as measured by both fluorescence intensity of lysates (Fig. 1C) and enumeration of T cells bound to the monolayers (Fig. 1D). In addition to the knockdown of MyoF, we obtained similar results comparing SLB-1 cells treated with WJ460, a small molecule inhibitor of MyoF (24), to vehicle control-treated cells (Fig. 1E and F). We had also established shMYOF and shGFP cells using ATL-2 cells (Fig. 1B)(12), an ATL patient-derived cell line. As with SLB-1 cells, knockdown of MyoF in ATL-2 cells reduced adhesion to HUVEC monolayers as determined from both the fluorescence of monolayer lysates and the number of T cells bound to intact monolayers (Fig. 2A and B). Together, these results indicate that MyoF promotes attachment of HTLV-1-infected T cells to endothelial cells.

### Knockdown of MyoF reduces HTLV-1-infected T-cell migration and invasion

**Fig 3:**
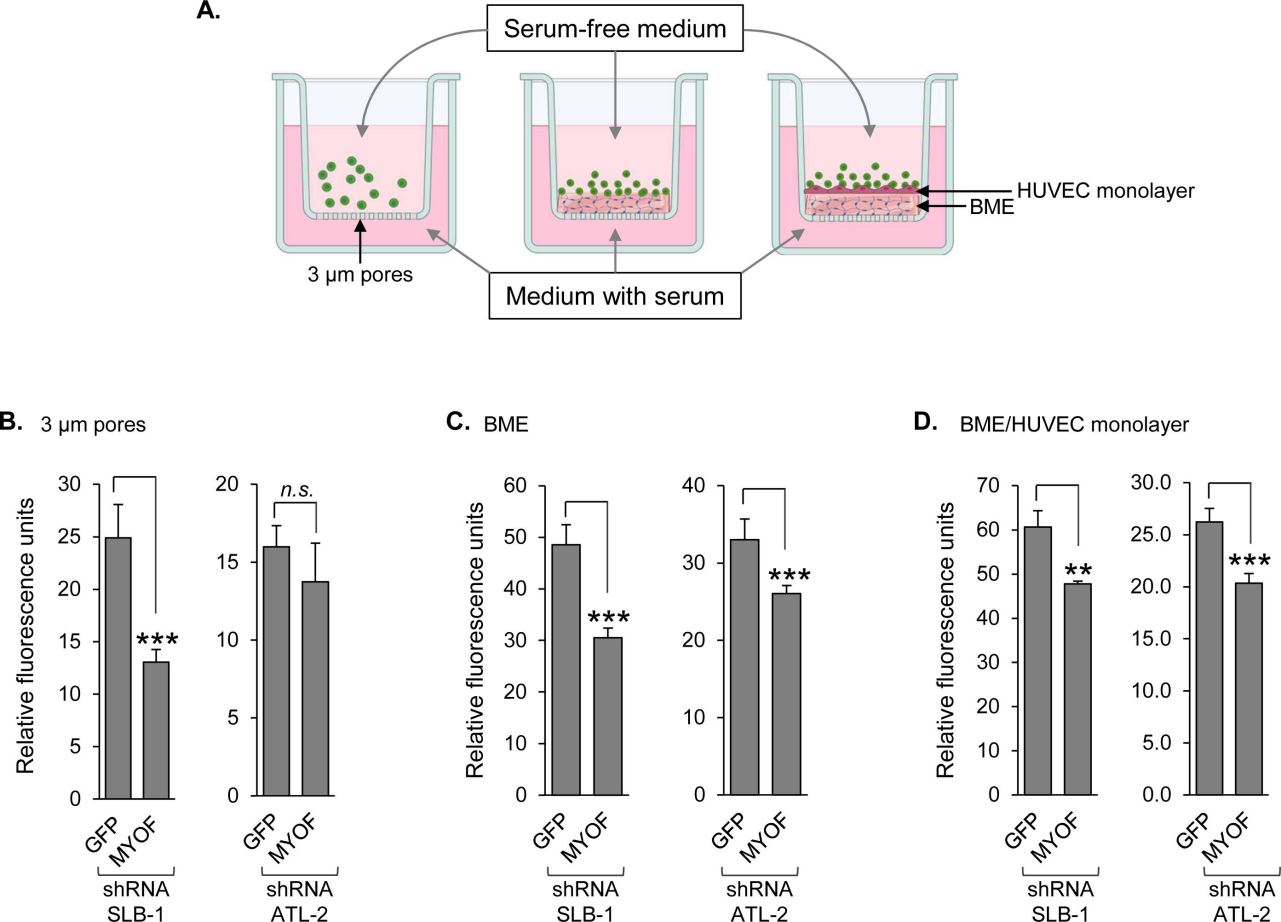
MyoF depletion reduces HTLV-1-infected T-cell migration and invasion. (**A**) The schematic shows the migration and invasion assays created using BioRender.com. SLB-1 and ATL-2 cells stably expressing an shRNA targeting GFP (negative control) or MYOF mRNA were serum-starved and fluorescently labeled (green). The cells in serum-free medium were then added to Boyden chambers (membrane alone; left) or Boyden chambers overlaid with basement membrane extract (BME; center) or with a HUVEC monolayer attached to a BME (right). Cells that reached the lower wells were lysed and analyzed for fluorescence. (**B**) Migration through Boyden chamber membranes alone within 1 h. (**C**) Migration through Boyden chambers overlaid with BME within 24 h. (**D**) Migration through Boyden chambers with a HUVEC monolayer within 24 h. Each graph shows fluorescence values of cell lysates averaged from three replicates of a single experiment and is representative of three independent experiments for SLB-1 and two for ATL-2; ** *P* < 0.01, ***P < 0.001 (*t*-test).

Based on the invasive properties of HTLV-1^+^ T cells and the role of MyoF in tumor cell metastasis (16, 28, 29), we investigated the possibility that MyoF contributes to the migration and invasion capacities of HTLV-1-infected T cells. To compare migration by shGFP and shMYOF cells established from both SLB-1 and ATL-2 lines, we used a Boyden chamber assay. Cells were cultured in serum-free medium, fluorescently labeled, and transferred to chambers with 3-µm pores in the membrane partitioning serum-rich medium in the wells (Fig. 3A). For SLB-1 lines, after 1 h, we found that significantly more shGFP cells had migrated to the lower chamber compared to shMYOF cells, while for ATL-2 lines, there was no significant difference in migration between shGFP and shMYOF cells (Fig. 3B). To then determine whether MyoF functions in HTLV-1-infected T-cell invasion, we conducted similar assays but overlaid the Boyden chamber membrane with Cultrex (mimics the basement membrane) alone or as a support for an activated HUVEC monolayer (Fig. 3A). Under both conditions after 24 h and with both SLB-1 and ATL-2 lines, significantly more shGFP cells were able to pass through the membrane (Fig. 3C and D), indicating that MyoF knockdown reduces invasiveness of these cells. Overall, these results suggest that MyoF enhances the migration and invasiveness of HTLV-1-infected T cells.

### MyoF regulates expression of genes involved in cell adhesion, migration, and invasion

**Fig 4:**
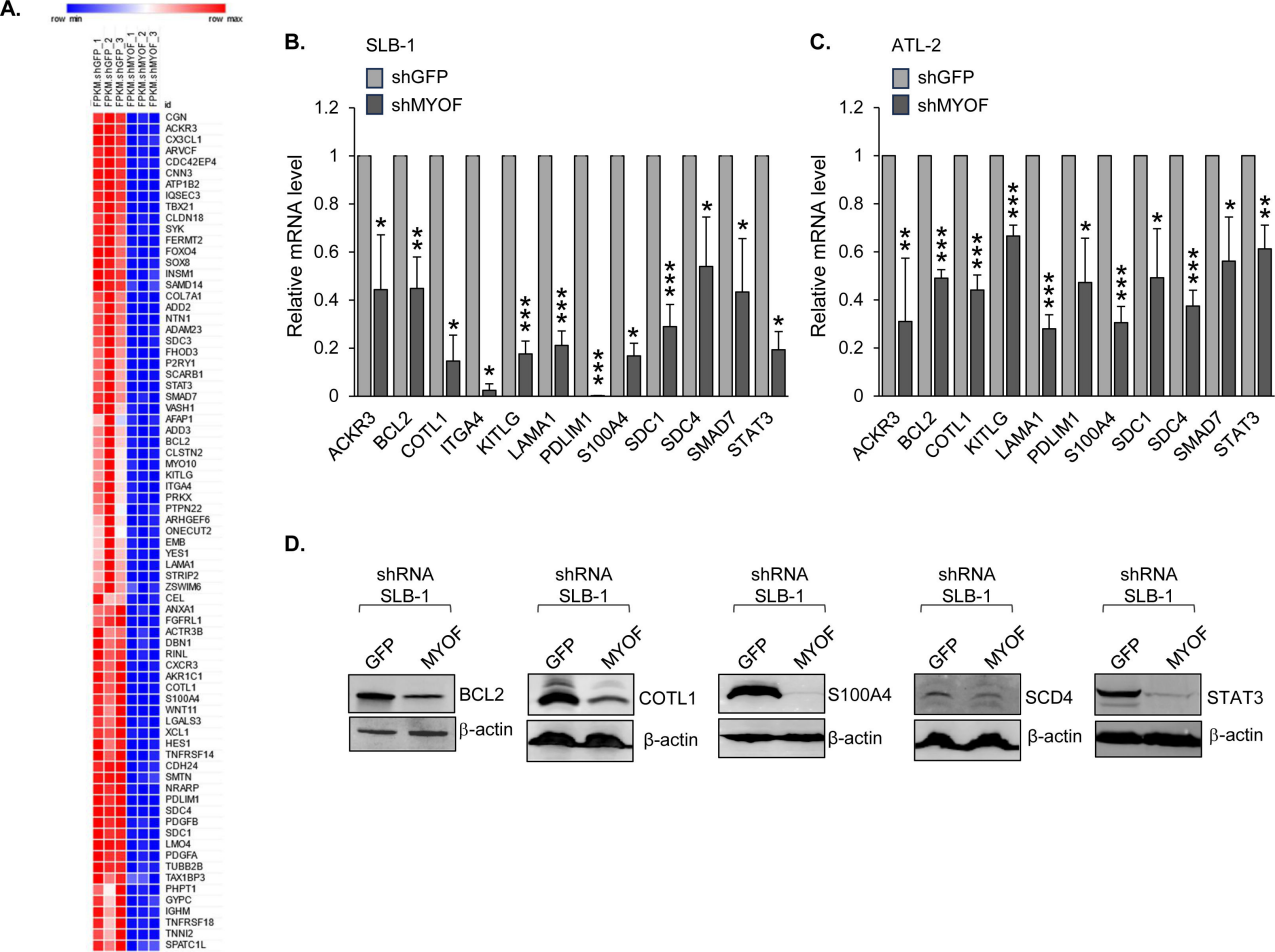
MyoF depletion affects cellular transcription. (**A**) The heat map shows the 75 genes involved in cell migration, cell adhesion, actin dynamics, or more than one of these processes, with lower levels of mRNA in SLB-1 cells stably expressing shRNA targeting MYOF mRNA (shMYOF) compared to the shGFP control cells. The heat map is limited to genes with FPKM values ≥ 1.95 in all shGFP replicates. (**B**) Relative ACKR3, BCL2, COTL1, ITGA4, KITLG, LAMA1, PDLIM1, S100A4, SDC1, SDC4, SMAD7 and STAT3 mRNA levels in SLB-1 shGFP versus shMYOF cells. The graph shows qRT-PCR results averaged from three independent experiments with values normalized to that of shGFP (set to 1) for each replicate (Mann-Whitney U test for COTL1, ITGA4, S100A4, and STAT3; *t*-test for other genes). (**C**) Relative ACKR3, BCL2, COTL1, KITLG, LAMA1, PDLIM1, S100A4, SDC1, SDC4, SMAD7, and STAT3 mRNA levels in ATL-2 shGFP versus shMYOF cells. The graph shows qRT-PCR results averaged from three independent experiments with values normalized to that of shGFP (set to 1) for each replicate (Mann-Whitney U test for PDLIM1; *t*-test for other genes); * *P* < 0.05, **P < 0.01, ***P < 0.001. (**D**) Protein expression of BCL2, COTL1, S100A4, SDC4, and STAT3 in SLB-1 shGFP and shMYOF cells. Whole cell extracts (BCL2: 35 μg; COTL1 and SDC4: 50 μg, S100A4: 80 μg, and STAT3: 40 μg) were analyzed by western blot using antibodies against the indicated proteins.

**Fig 5:**
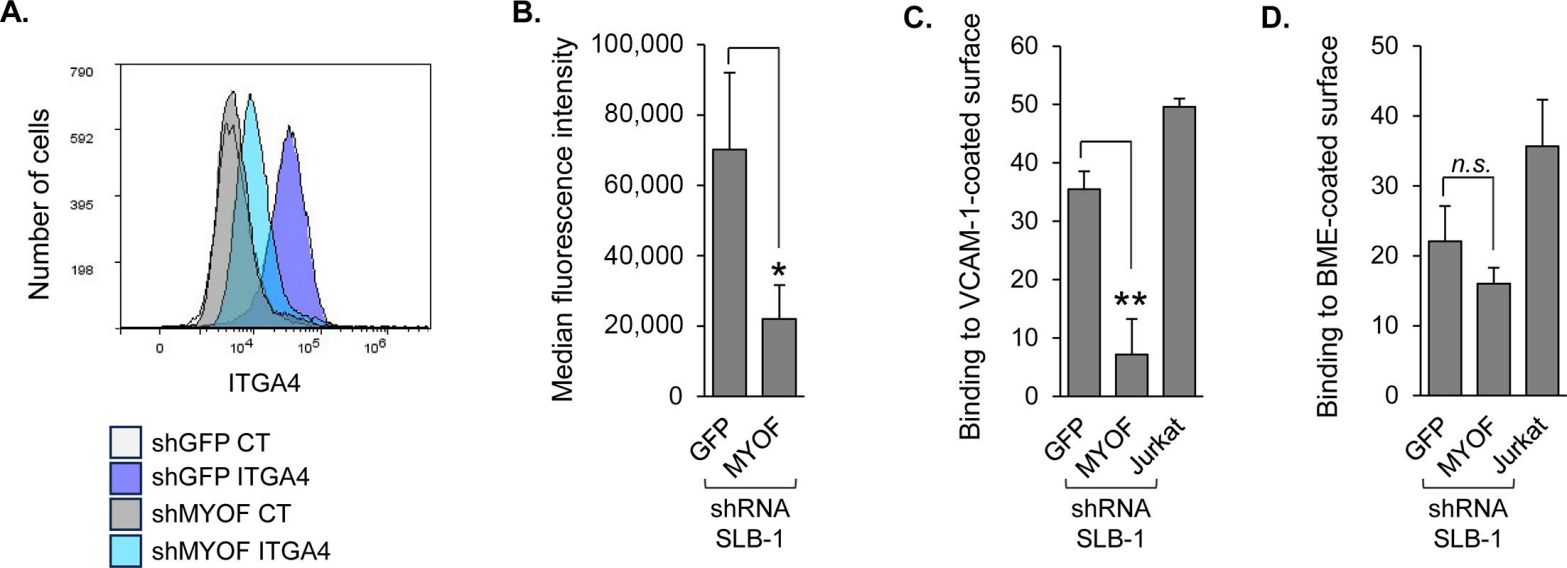
MyoF depletion reduces binding of HTLV-1-infected T cells to VCAM-1. (**A**) Knockdown of MyoF expression reduces the level of integrin α4 (ITGA4) at the cell surface. SLB-1 cells stably expressing shRNA targeting GFP (shGFP) or MYOF mRNA (shMYOF) were labeled with an antibody against integrin α4, followed by a FITC-conjugated secondary antibody, fixed, and analyzed by flow cytometry. The histograms (representative of three independent experiments) show relative cell surface labeling as follows: control (CT) shGFP and shMYOF cells labeled with secondary antibody alone are white and gray, respectively; ITGA4-labeled shGFP and shMYOF cells are lavender and light blue, respectively. (**B**) The graph shows the mean fluorescence intensities of cell-surface integrin α4 for shGFP and shMYOF cells averaged from three independent experiments; * *P* < 0.05 (*t*-test). (**C**) Knockdown of MyoF expression reduces binding to VCAM-1. (**D**) Knockdown of MyoF does not affect binding to Cultrex. Jurkat cells and SLB-1 cells stably expressing shRNA targeting GFP (shGFP) or MYOF mRNA (shMYOF) were labeled with Calcein AM and analyzed for binding to VCAM-1 or Cultrex immobilized in 96-well plates. The graph shows fluorescence values averaged from three replicates for each cell type in a single experiment and is representative of three independent experiments; ** *P* < 0.01 (*t*-test).

MyoF affects signaling pathways through regulation of receptor trafficking and protein stability (16, 17), which produces downstream effects on gene expression. To identify genes with expression affected by MyoF, we performed an RNA-seq analysis, comparing SLB-1 shGFP and shMYOF cells. The results revealed 2,048 genes with a significant reduction in expression in shMYOF cell lines, and from this group, protein-coding genes were selected with fragments per kilobase per million reads mapped (FPKM) values ≥ 1.95 in all shGFP replicates (Table S1). In the resulting pool of 402 genes, 41, 32, and 33 genes were associated with cell migration, cell adhesion, and actin dynamics, respectively, with some genes involved in more than one of these processes (Fig. 4A). Quantitative RT-PCR (qRT-PCR) was used to verify RNA-seq data from a subset of genes involved in one or more of the three biological processes. Specifically, reduced expression in SLB-1 shMYOF cells compared to shGFP cells was confirmed for *ACKR3*, *BCL2*, *COTL1*, *ITGA4*, *KITLG*, *LAMA1*, *PDLIM1*, *S100A4*, *SDC1*, *SDC4, SMAD7,* and *STAT3* (Fig. 4B). qRT-PCR analysis of these genes in ATL-2 shGFP and shMYOF cells revealed a similar trend, but with generally less dramatic reductions in expression (Fig. 4C). However, surprisingly, ATL-2 cells were found to be unique in that they lacked *ITGA4* expression. Within the group of tested genes, protein levels of Bcl-2, coactosin-like F-actin binding protein 1 (*COTL1* gene), S100A4, syndecan 4 (*SDC4* gene), and STAT3 were confirmed to be reduced in SLB-1 shMYOF cells compared to shGFP cells (Fig. 4D). Given a weak signal, syndecan 4 protein levels were quantified to verify its reduction with MYOF knockdown (Fig. S2C). In addition, integrin α4 (*ITGA4* gene) was confirmed to be lower on the surface of SLB-1 shMYOF cells compared to shGFP cells (Fig. 5A and B). However, as expected based on the qRT-PCR results, no integrin α4 was detected on the ATL-2 cells. Integrin α4 heterodimerizes with integrin β1, forming the VLA-4 complex (32). Expression of the genes encoding integrin β1 was similar between shGFP and shMYOF cells (Fig. S2A). VLA-4 binds VCAM-1 on the surface of endothelial cells (32), and consistent with a lower level of surface integrin α4, shMYOF cells displayed a lower capacity to adhere to a VCAM-1-coated surface than shGFP cells (Fig. 5C). In contrast, there was no significant difference in the adhesion of shGFP versus shMYOF cells to a Cultrex-coated surface (Fig. 5D).

These proteins were specifically selected for analysis based on their association with ATL or HTLV-1-transformed T-cell adhesion and migration (ITGA4 [33], SDC4 [34], COTL1 and S100A4 [35], BCL2 [36], and STAT3 [37–39]). Among the genes encoding these proteins, analysis of a published microarray data set (40) revealed that expression of COTL1 and S100A4 was significantly elevated in CD4^+^ T cells from acute ATL patients compared to CD4^+^ T cells from healthy donors (Fig. S3). While S100A4 has been shown to consistently promote cancer cell migration and metastasis (41–43), COTL1 has been reported to play both positive and negative roles in these processes (44–46). This discrepancy prompted us to characterize the role of COTL1 in HTLV-1-infected T cells.

### MyoF, but not COTL1, can affect the size of HTLV-1-infected T cells

**Fig 6:**
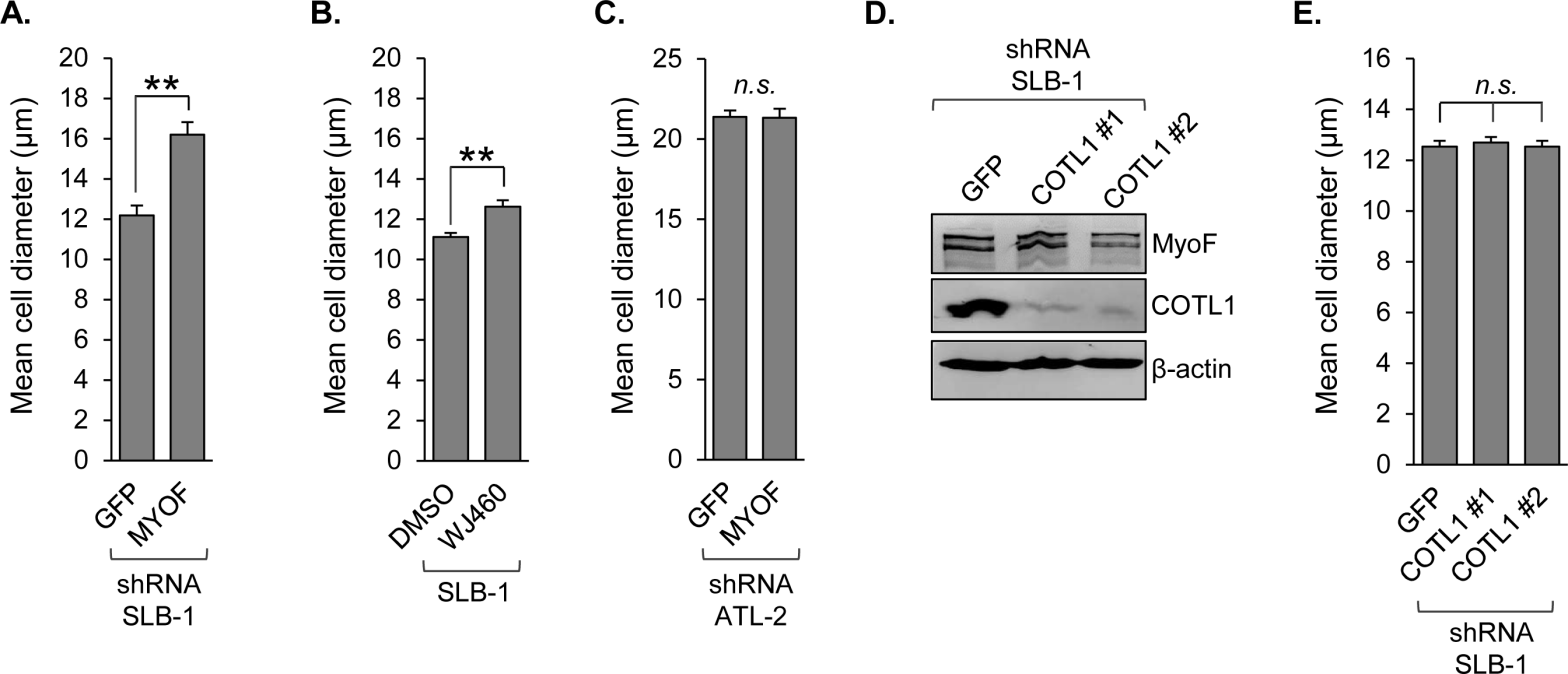
Inhibition or depletion of MyoF increases the size of HTLV-1-infected T cells. (**A**) Mean cell diameters of SLB-1 cells stably expressing shRNA targeting GFP or MYOF mRNA, and (**B**) SLB-1 cells treated with DMSO or 1 μM WJ460 for 24 h. Graphs show mean values of three reads from each culture and are representative of two independent experiments; ** *P* < 0.01 (*t*-test). (**C**) Mean cell diameters of ATL-2 cells stably expressing shRNA targeting GFP or MYOF mRNA. (**D**) COTL1 expression in SLB-1 cells stably expressing shRNA targeting GFP or COTL1; two COTL1 knockdown cell lines were established that express different shRNAs (shCOTL1 #1 and #2). Whole cell extracts (50 μg) were analyzed by western blot using antibodies against MyoF, COTL1, and β-actin. (**E**) Mean cell diameter of SLB-1 cells stably expressing shRNA targeting GFP (shGFP) or COTL1 mRNA.

Previous work with intestinal epithelial cells (IECs) revealed a positive role for COTL1 in extracellular matrix attachment, cell-cell adhesion, and cell migration, and also identified a role for COTL1 in regulating cell size (47). Interestingly, similar to the knockdown of COTL1 in IECs, the knockdown of MyoF in SLB-1 cells led to an increase in cell size (Fig. 6A; Fig. S4A). Furthermore, functional inhibition of MyoF with WJ460 also caused an increase in cell size (Fig. 6B). In contrast, knockdown of MyoF in ATL-2 cells, which are larger than SLB-1 cells, did not lead to an increase in cell size (Fig. 6C). To analyze its effects in HTLV-1-infected T cells, COTL1 was stably knocked down in SLB-1 cells using two different lentiviral vectors to generate shCOTL1 #1 and shCOTL1 #2 cells; control shGFP cells were also concomitantly established (Fig. 6D). Surprisingly, we did not detect a significant difference in the sizes of shCOTL1 versus shGFP cells (Fig. 6E; Fig. S4B). These results suggest that the MyoF-mediated expression of COTL1 is not sufficient to regulate cell size, and other events controlled by MyoF are required.

### MyoF and COTL1 increase levels of F-actin in HTLV-1-infected T cells

**Fig 7:**
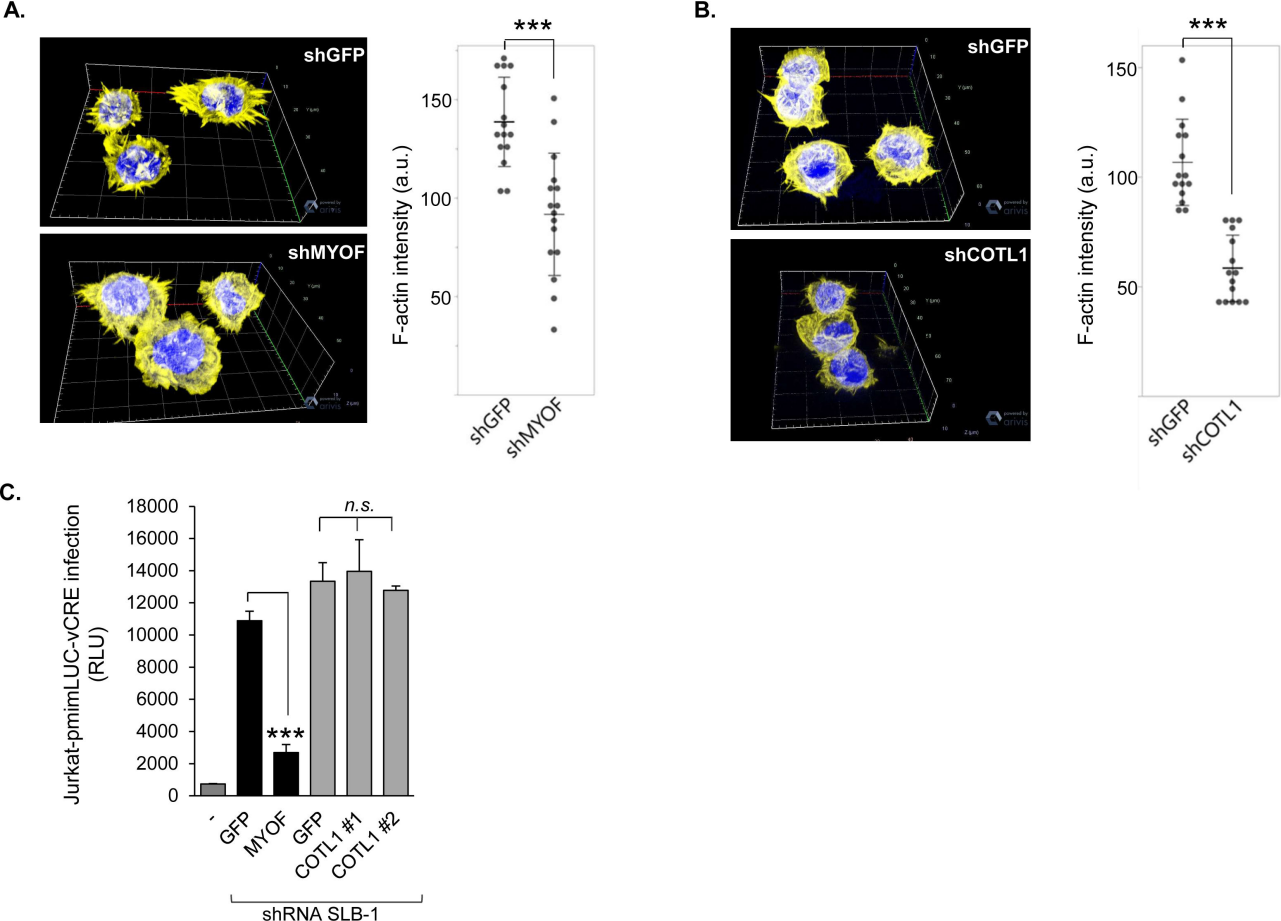
MyoF and COTL1 depletion reduce levels of F-actin in HTLV-1-infected T cells. (**A**) Knockdown of MyoF expression reduces F-actin. SLB-1 cells stably expressing an shRNA targeting GFP or MYOF mRNA were fixed, permeabilized, labeled with phalloidin (yellow) and DAPI (blue), and analyzed by confocal microscopy. The images show 3D projections constructed from z-stacks comprised of 0.6 μm optical slices. The graph shows F-actin intensity, *n* = 15 (shGFP) and *n* = 16 (shMYOF) from a single experiment and is representative of three independent experiments; *** *P* < 0.001 (*t*-test). (**B**) Knockdown of COTL1 expression reduces F-actin. SLB-1 cells stably expressing an shRNA targeting GFP or *COTL1* mRNA (shCOTL #1) were processed as described for panel A. The images show 3D projections constructed from z-stacks comprised of 0.4-μm optical slices. The graph shows F-actin intensity, *n* = 15 (shGFP) and *n* = 15 (shCOTL1) from a single experiment and is representative of two independent experiments; ***P < 0.001 (Mann-Whitney U test). (**C**) SLB-1 cells stably expressing shRNA targeting MYOF mRNA and the corresponding GFP control (black bars), or COTL1 mRNA and corresponding GFP control (gray bars) were co-cultured with Jurkat-pminLUC-vCRE cells. The graph shows luciferase assay results averaged from three replicates for each infection condition of a single experiment and is representative of three independent experiments; ***P < 0.001 (*t*-test).

Formation of F-actin structures plays an important role in cell adhesion, migration, and invasion (48). To compare levels of F-actin between paired shGFP and shMYOF cells, we fixed and stained cells with phalloidin. Using confocal microscopy, we observed a reduction in the F-actin signal intensity with knockdown of MyoF (49) (Fig. 7A). We performed identical experiments with paired shGFP and shCOTL1 cells and observed that the F-actin signal intensity was also decreased with knockdown of COTL1 (Fig. 7B). Given that, in T cells, COTL1 contributes to lamellipodia formation at the immunological synapse (50), we reasoned that COTL1 might also contribute to formation of the virological synapse and thus HTLV-1 infection. However, in coculture infection assays, knockdown of COTL1 in the SLB-1 effector cells did not affect the level of viral infection as deduced by luciferase activity from the Jurkat pminLUC-vCRE reporter/target cells (11) (Fig. 7C). Consistent with our previous results (12), knockdown of MyoF did reduce viral infection. Overall, these results suggest that effects of MyoF on F-actin occur, in part, through COTL1; however, the regulation of F-actin structures by COTL1 does not appear to play a significant role in HTLV-1 infection.

### Knockdown of COTL1 increases HTLV-1-infected T-cell adhesion to endothelial cells but reduces HTLV-1-infected T-cell migration and invasion

**Fig 8:**
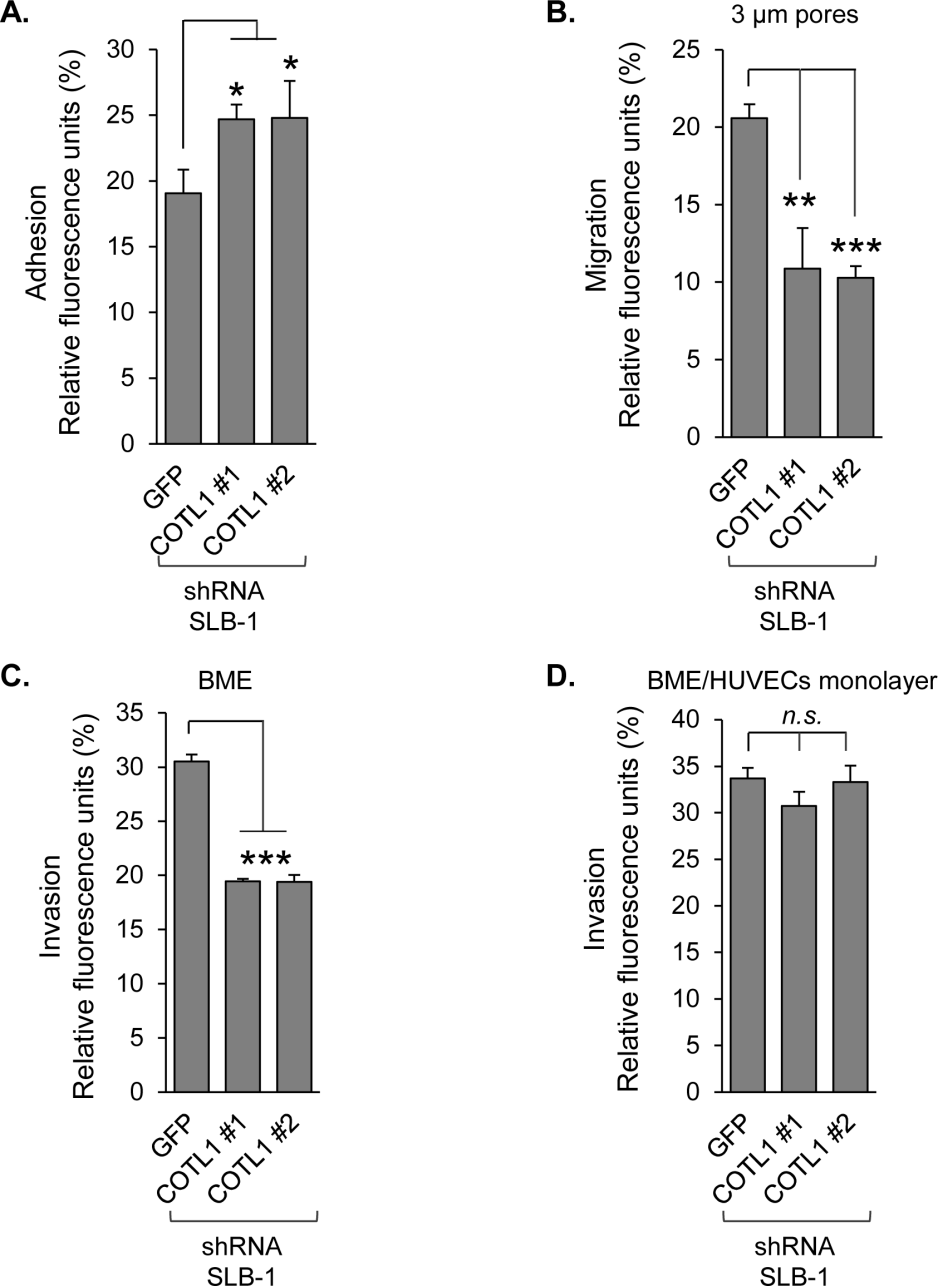
COTL1 depletion reduces HTLV-1-infected T-cell migration and invasion through BME. (**A**) Calcein AM-labeled SLB-1 cells stably expressing an shRNA targeting GFP (negative control) or COTL1 (shCOTL1 #1 and #2) mRNA were cultured on HUVEC monolayers and then lysed and analyzed for fluorescence. The graph shows fluorescence values averaged from three replicates of a single experiment and is representative of two independent experiments; *P < 0.05 (*t*-test). (**B–D**) SLB-1 cells stably expressing an shRNA targeting GFP (negative control) or COTL1 (shCOTL1 #1 and #2) mRNA were serum-starved and fluorescently labeled. The cells in serum-free medium were then added to Boyden chambers (membrane alone) or Boyden chambers overlaid with basement membrane extract (BME) or with a HUVEC monolayer attached to a BME. Cells that reached the lower wells were lysed and analyzed for fluorescence. Migration through Boyden chamber membranes alone within 1 h (**B**). Migration through Boyden chambers overlaid with BME within 24 h (**C**). Migration through Boyden chambers with a HUVEC monolayer within 24 h (**D**). Each graph shows fluorescence values of cell lysates averaged from three replicates from a single experiment and is representative of two independent experiments; **P < 0.01, ***P < 0.001 (*t*-test).

The similar effects of MyoF and COTL1 on F-actin levels in HTLV-1-infected T cells and the involvement of actin dynamics in cell adhesion, migration, and invasion led us to test whether COTL1, like MyoF, influences these processes. In comparing the ability of shGFP and shCOTL1 cells (labeled with Calcein AM) to bind a HUVEC monolayer (Fig. 1A), we found that in contrast to the knockdown of MyoF, the knockdown of COTL1 led to an increase in endothelial cell adhesion (Fig. 8A). For these assays, we verified that there was no difference in labeling between shGFP and shCOTL1 cells (Fig. S1B) and no change in ITGA4 expression (Fig. S2B). Cell migration was analyzed by comparing the ability of shGFP and shCOTL1 cells (serum-starved and fluorescently-labeled) to traverse the 3-µm pores of a Boyden chamber membrane to the serum-rich chamber (Fig. 3A). The results from these assays showed that knockdown of COTL1 produces similar effects as knockdown of shMYOF, reducing the capacity of cells to cross the membrane within a 1 h experimental time frame (Fig. 8B). To analyze cell invasion, we first compared the ability of shGFP and shCOTL1 cells (serum-starved and fluorescently labeled) to cross a Cultrex matrix overlaid on the Boyden chamber membrane. Similar to the knockdown of MyoF, the knockdown of COTL1 reduced the ability of cells to penetrate the basement membrane-like matrix (Fig. 8C). We also compared the ability of shGFP and shCOTL1 cells (serum-starved and fluorescently labeled) to penetrate a HUVEC monolayer. Unlike the knockdown of MyoF, the knockdown of COTL1 did not affect transendothelial cell migration. These results suggest that the role of COTL1 may be restricted to facilitating HTLV-1-transformed T-cell migration through extracellular matrices.

## DISCUSSION

In this study, we provide evidence that expression of MyoF in HTLV-1-transformed T cells enhances cell adhesion and invasion properties. These properties are in line with the ability of HTLV-1-infected cells to infiltrate the central nervous system in HAM/TSP patients and separately relate to malignant cell infiltration of the skin, visceral organs, and bone associated with ATL (28, 51). Our previous analysis of published gene expression microarray data indicated that *MYOF* expression is substantially higher in CD4^+^ T cells from ATL patients than those from HAM/TSP patients (12). Therefore, the contribution of MyoF to infected T-cell adhesion, migration, and invasiveness may be more pronounced in ATL. Consistent with this premise, MyoF is overexpressed in several epithelial cancers, leading to an overall poor patient outcome, in part, by promoting tumor cell metastasis (21, 23–27).

Here, we analyzed the effects of MyoF using two HTLV-1-transformed T-cell lines: SLB-1 and ATL-2. Knockdown of MyoF in both cell lines led to a reduction in adhesion to an endothelial monolayer as well as reductions in invasion through a reconstituted basement matrix and through an endothelial monolayer. However, while knockdown of MyoF in SLB-1 cells reduced migration through 3-µm pores of the Boyden chamber membrane, knockdown of MyoF in ATL-2 cells did not. Possibly related to this difference is our observation that ATL-2 cells are larger than SLB-1 cells, and unlike SLB-1 cells, knockdown of MyoF in ATL-2 cells did not lead to an additional increase in cell size. Therefore, cell size may be a major factor in the migration assay. These results underscore that the effects of MyoF in SLB-1 cells were not fully recapitulated in ATL-2 cells. Also, potentially relevant to this issue is the fact that, in ATL-2 cells, MyoF expression is lower.

Activation of MyoF expression complements other effects of HBZ that promote HTLV-1-infected T-cell migration and invasion. Indeed, HBZ activates expression of the transcription factor, GATA3, which in turn activates expression of CCR4 (52), a chemokine receptor that is overexpressed in ATL cells (53). In HBZ-transgenic mice, CCR4 was found to increase the migration of CD4^+^ T cells (52). In a separate study, HBZ was also shown to induce expression of noncanonical Wnt5a, which facilitated migration of HTLV-1-transformed T cells (54). It is possible that the use of multiple mechanisms by HBZ enforces its ability to promote cell migration and invasion.

Our RNA-seq analysis supports that, in HTLV-1-transformed T cells, the ability of MyoF to augment cell adhesion, migration, and invasive properties stems, in part, from its influence on the expression of genes involved in these processes. While MyoF does not function as a *bona fide* transcriptional regulator, it may affect gene expression by modulating receptor recycling and, in turn, autocrine and paracrine signaling that culminate in transcriptional changes (16, 55–57). MyoF also affects mitochondrial dynamics (18, 58–61), which, through production of reactive oxygen species, can impact transcription (62). In a more direct role, MyoF has been found to transport activated STAT3 into the nucleus following IL-6 stimulation (63). Based on these observations, the expression of distinct subsets of genes may be influenced by MyoF through different mechanisms. Further work is needed to address this hypothesis.

The initial processes required for infiltration of T cells into tissues and organs are extravasation, followed by cell migration through the basement membrane and interstitium (30). Extravasation fundamentally involves cell adhesion to the endothelium followed by cell crawling, leading to transendothelial migration (32). Results from our *in vitro* assays indicate that MyoF contributes to extravasation, and our RNA-seq and follow-up protein analysis indicate that expression of integrin α4 may play a role in this process. On T cells and other leukocytes, integrin α4 heterodimerizes with integrin β1 to form VLA-4, which binds VCAM-1 on endothelial cells, enabling firm adhesion and crawling (32). Furthermore, VLA-4 binds junctional adhesion molecule 2 on endothelial cells, which may facilitate transendothelial migration (64). Consistent with our findings, expression of VLA-4 has been reported to be high in HTLV-1^+^ T cells, including cells from both HAM/TSP and ATL patients, and has been implicated in the adhesion properties of these cells (33, 65–68). Furthermore, while unchanged in our RNA-seq data, *ITGB1* (which encodes integrin β1) is reported to be a driver gene for ATL (69).

While VLA-4 potentially contributes to early events during extravasation of HTLV-1^+^ T cells, it may not be essential for this process. Surprisingly, we found that ATL-2 cells are atypical in that they do not express integrin α4. However, knockdown of MyoF still led to a reduction in binding of ATL-2 cells to an endothelial monolayer, suggesting the involvement of additional adhesion molecules in this process. Cell adhesion molecule 1 (CADM1), L-selectin, and OX40 have been reported to be highly expressed in ATL cells and are implicated in promoting endothelial adhesion during extravasation of ATL cells (70–72). While our RNA-seq data indicated similar CADM1 expression levels between shGFP and shMYOF, OX40 expression trended lower in shMYOF cells but was not statistically significant. In contrast, L-selectin (*SELL* gene) expression was significantly lower in shMyoF. However, due to its overall low level of expression, we did not pursue *SELL*. Considering possible variations in gene expression between SLB-1 and ATL-2 cells, OX40 and L-selectin may be more relevant to MyoF-mediated endothelial cell adhesion for ATL-2 cells. Alternatively, MyoF may influence endosomal trafficking and, in turn, cell surface abundance of certain adhesion molecules, which will not be evident from RNA-seq data.

From the RNA-seq data, we also confirmed that protein levels of Bcl-2, syndecan 4, STAT3, S100A4, and COTL1 were reduced with the knockdown of MyoF, suggesting that MyoF has a positive effect on the expression of these proteins. These proteins contribute to various aspects of cancer cell biology, including cell migration and invasion, implying their relevance to the infiltration capabilities of HTLV-1^+^ T cells. Bcl-2 and related family members, in addition to preventing apoptosis, stimulate cell invasion through a variety of mechanisms (73), and in the context of ATL, inhibition of these proteins was shown to reduce tumor growth in a mouse model (36). Syndecan 4 is involved in multiple cancer cell processes, including cell migration (74). Consistent with this effect, knockdown of syndecan 4 in an HTLV-1-transformed T-cell line was shown to cause a significant decrease in *in vitro* cell migration (34). STAT3 signaling has pleiotropic effects on cancer biology (75), and in some cancers, STAT3 expression is upregulated (76). While an increase in STAT3 expression in ATL has not been reported, an analysis from one gene expression microarray data set revealed higher expression of *STAT3* in CD4^+^ T cells from chronic ATL donors compared to those from uninfected donors (Fig. S3C). Importantly, the authors of this study used a cell sorting strategy to enrich HTLV-1-infected CD4^+^ T cells from the total pool of CD4^+^ T cells (77). However, in the context of HTLV-1 infection, transition to constitutive activation of STAT3 in HTLV-1^+^ T cells is associated with cell transformation as well as proliferation of ATL cells (37, 38). Furthermore, an analysis of ATL cells from patients revealed that STAT3 accumulates somatic mutations associated with its constitutive activation (39). S100A4 is well known to drive cancer cell metastasis through a variety of mechanisms (78), while COTL1 has been implicated in both antagonizing and promoting cancer cell functions (44–46, 79). In a study characterizing the pathways responsible for the transition of patient HTLV-1-infected cells into leukemic cells, *COTL1* and *S100A4* were identified along with 36 other genes consistently exhibiting differential expression in this process (35).

In epithelial cancer cells, MyoF stimulates migration and invasion by facilitating epithelial-mesenchymal transition (EMT), resulting in the release of cells from the tumor, adoption of an elongated morphology, and expression and activation of matrix metalloproteases (MMPs) (20, 21, 23–25, 27). These changes allow cells to degrade and thereby penetrate through the extracellular matrices during metastasis. This mesenchymal mode of motility is also aided by focalized, stable integrin-mediated binding to extracellular matrices (80). We do not believe that MyoF produces similar effects in HTLV-1-transformed T cells, as according to our RNA-seq data, knockdown of MyoF did not decrease transcripts of MMPs associated with EMT, such as MMP-2 and -9, or other transcripts associated with the EMT gene expression signature, such as the genes encoding Snail 1 and 2 and Zeb 1 and 2. Instead, MyoF may promote processes important for amoeboid migration through extracellular matrices typical of leukocytes and leukemic cells (80).

Unlike mesenchymal motility, amoeboid motility does not require the formation of focal adhesion contacts with the extracellular matrix or matrix degradation but rather relies on dynamic morphological changes and contractile forces that allow cells to navigate through constricted spaces, such as those presented by extracellular matrices (81). These processes are, in part, modulated by remodeling of cortical actin and actomyosin forces generated from the uropod of the cell and transmitted to the cell cortex (82). We believe MyoF contributes to amoeboid motility of HTLV-1-transformed T cells, as MyoF knockdown leads to a reduction in *in vitro* cell migration through a basement membrane-like matrix. This outcome may be associated with the effect of MyoF on STAT3, S100A4, and COTL1 expression. Activated Stat3 has been shown to promote amoeboid motility of both melanoma and diffuse large B-cell lymphoma cells (83, 84). This function involves crosstalk with the Rho-ROCK signaling pathway, which regulates non-muscle myosin II activity and thus actomyosin contractility (85). Interestingly, in stimulating cell motility and invasiveness, S100A4 also targets non-muscle myosin II (86–88), specifically by binding to non-muscle myosin heavy chain IIA (NMIIA) (89). This interaction destabilizes myosin II filaments and inhibits their formation (90), which, in macrophages, promotes cell migration by preventing over-assembly of NMIIA (91). Overall, it is possible that MyoF enhances HTLV-1^+^ T-cell migration and invasion through the combined effects of S100A4 and STAT3 coordinating actomyosin contractility.

Our results indicate that COTL1 expression through MyoF is also important for HTLV-1-transformed T-cell motility. Indeed, we found that knockdown of COTL1 mimicked the effects of knockdown of MyoF on *in vitro* cell migration and invasion, inhibiting both processes. Similar effects of COTL-1 knockdown were previously shown for Jurkat T cells (50). Surprisingly, we found that COTL1 knockdown cells display an increase in endothelial cell adhesion, suggesting that COTL1 inhibits HTLV-1-transformed T-cell interactions with endothelial cells. In contrast, MyoF produced the opposite effect, enhancing interactions with endothelial cells. Given that MyoF influences a broad range of cellular processes (16, 92), this discrepancy may be due to MyoFs overshadowing the effect of COTL1 on adhesion. It is likely that COTL1 contributes to HTLV-1-transformed T-cell migration and invasiveness through its ability to bind and stabilize F-actin (50). In a DLD1 colonic epithelial cell monolayer, knockdown of COTL1 was found to disrupt the actomyosin bundles of the peri-junctional cytoskeletal structure (47). In applying this observation to HTLV-1-transformed T cells, it is possible that, as proposed for S100A4 and STAT3, COTL1 regulates actomyosin contractility to promote amoeboid motility. Future studies will test this hypothesis.

The specific mechanism through which MyoF increases COTL1 transcription, specifically, is unclear. Recently, RNA-seq data for MyoF knockdown in pancreatic ductal adenocarcinoma cells was made available (93). An analysis of these data sets showed no change in COTL1 expression with MyoF knockdown, suggesting that the effects of MyoF on COTL1 transcription might be specific to HTLV-1-transformed T cells, which might relate to specific autocrine/paracrine signaling pathways active in the infected cells. Further work is needed to characterize how MyoF affects COTL1 transcription in HTLV-1-transformed cells and how MyoF affects transcription in general.

## MATERIALS AND METHODS

### Cell culture and generation of cell lines

Jurkat pminLUC-viral CRE, SLB-1, and ATL-2 cells were cultured in Iscove’s modified Dulbecco medium (IMDM). SLB-1 (S-LBI) cells were established by coculturing terminally irradiated ME cells, established from an adult T-cell leukemia patient, with peripheral blood from a female donor (94). Jurkat pminLUC-viral CRE and SLB-1 and ATL-2 shGFP and shMYOF cells were established previously (11, 12). These cells were supplemented with 10% fetal bovine serum (FBS) or 10% Fetalplex animal serum (Gemini Bio-Products) and 2 mM l-glutamine, 100 U/mL penicillin, and 50 μg/mL streptomycin. Human umbilical vein endothelial cells (HUVECs) were cultured in Endothelial Cell Growth Media (CCM027, R&D Systems). SLB-1 shCOTL1 and paired shGFP cells were established by lentivirus transduction as described in a previous study (12). COTL1 shRNA MISSION plasmids used were TRCN0000291958 and TRCN0000292021 (MilliporeSigma).

### Western blotting

Cells (5 × 10⁵ cells/mL) were cultured overnight and then harvested. Whole cell extracts were prepared, and western blotting was performed as described (95) using the following antibodies: anti-MYOF (HPA14245; Millipore Sigma), anti-β-actin (sc-47778), anti-Bcl-2 (sc-492), anti-STAT3 (sc-8019), anti-SDC4 (sc-12766), and anti-S100A4 (sc-377059) were purchased from Santa-Cruz and anti-COTL1 (PA5-122072; Invitrogen). Blots were developed using Pierce ECL Plus (Thermo Fisher Scientific) and visualized with a Typhoon RGB imager (Cytiva). Image analysis was performed using ImageQuant TL v8.1 software (GE Healthcare Lifesciences).

### Adhesion assays

HUVECs were seeded at 5 × 10⁵ cells per well in 24-well plates pre-coated with 0.1% gelatin, grown to 80% confluency (48–72 h), and activated with 10 ng/mL TNF-α (Cell Biolabs, Inc.) for 6 h. Adhesion assays were performed as described previously (12). A control well contained Calcein AM-labeled cells that did not undergo manipulations was used to calculate the percent fluorescent signal as follows: (fluorescence of experimental wells)/(fluorescence of control well) × 100.

### Migration assays

Cells stably expressing shRNA vectors (5 × 10^5^ cells/mL) were cultured overnight in serum-free medium and then centrifuged and resuspended in PBS (137 mM NaCl, 2.7 mM KCl, 3 mM Na_2_HPO_4_, and 1.5 mM KH_2_PO_4_) to 1 × 10^6^ cells/mL. Cells were then stained with 1X LeukoTracker Solution (Cell Biolabs INC.) for 1 h at 37°C, protected from light. Following staining, cells were centrifuged, resuspended in pre-warmed serum-free medium to 1 × 10^6^ cells/mL and incubated for 10 m at 37°C/5% CO_2_. Cells (1.2 × 10^5^) were then added to transwell inserts (3-μm pore size) that were placed in wells (24-well plate) containing 0.5 mL of medium with 10% FBS and incubated for 1 h at 37°C/5% CO_2_. Inserts with the medium and non-migrated cells were removed from the wells to which 150 μL of 4× Passive Lysis Buffer (Promega) was added. Cells were lysed by mechanical shaking for 5 min. Cell lysates (150 μL) were transferred to 96-well plates, and fluorescence intensity was measured at 488/520 nm using a Varioskan LUX plate reader with SkanIt 7.1 software (ThermoFisher Scientific).

### BME invasion assays

Cells were prepared, and assays were conducted as described for “Migration assays,” above, but with the following two modifications. First, prior to labeling the cells, transwell inserts (3 μm pore size) were coated with 100 µL of Cultrex Basement Membrane Extract (BME; R&D Systems) diluted 1:20 in cold serum-free medium for 1 h at room temperature. Second, once the coated inserts with the cells were placed in wells, the 37°C/5% CO_2_ incubation proceeded for 24 h.

### HUVEC invasion assays

Transwell inserts (3 μm pore size) were coated with 100 µL of Cultrex BME diluted 1:100 in cold serum-free medium for 1 h at room temperature. HUVECs were seeded at 100,000 cells per coated insert and cultured for 48-72 hours until reaching approximately 80% confluency. HUVECs were then activated with 10 ng/mL of TNF-α (Cell Biolabs, Inc.) for 6 h. HTLV-1^+^ T-cells were prepared, and assays were conducted as described for “Migration assays” and “BME invasion assays,” above, using a 24 h incubation time for transmigration. A single modification to the assays involved the lysis step: cells in the insert were removed with a cotton swab, and 150 μL of 4× Passive Lysis Buffer was added to swabbed inserts, which were placed back into the wells. Subsequent steps were the same as described for “Migration assays,” above.

### RNA-seq

SLB-1 shGFP and shMYOF cells (5 × 10^5^ cells/mL) were cultured overnight. Cells were then harvested and RNA isolated using the Total RNA Purification Kit (Norgen Biotek). Three biological replicates were produced. RNA specimens were processed by LS Sciences as follows. RNA integrity was verified using a 2100 Bioanalyzer System (Agilent Technologies). Poly(A) tail-containing mRNA was purified using oligo-(dT) magnetic beads with two rounds of purification. Purified, poly(A) RNA was fragmented using a divalent cation buffer at elevated temperature, and a Poly(A) RNA sequencing library was prepared using a TruSeq Stranded mRNA kit (Illumina) according to the manufacturer’s instructions. Quality control analysis and quantification of the sequencing library were performed using a 2100 Bioanalyzer High Sensitivity DNA Chip (Agilent Technologies). Paired-end sequencing was performed using a NovaSeq 6000 Sequencing System (Illumina). Downstream analysis was done by LS Sciences. First, reads with low quality and undetermined bases and reads with adapter contamination were removed using Cutadapt (96) and in-house Perl scripts. The sequence quality was then verified using FastQC (Babraham Bioinformatics), and reads were mapped to the genome (ftp://ftp.ensembl.org/pub/release-101/fasta/homo_sapiens/dna/) using HISAT2 (97). Mapped reads for each specimen were assembled using StringTie (98), and specimen transcriptomes were merged to reconstruct a comprehensive transcriptome using Perl scripts and gffcompare. Expression levels for all transcripts were estimated using StringTie (98) and ballgown (http://www.bioconductor.org/packages/release/bioc/html/ballgown.html). Differential mRNA expression between two samples and two groups was determined using edgeR and DESeq2, respectively (99, 100). mRNA with false discovery rates (FDR) below 0.05, and absolute fold changes ≥ 2 were considered differentially expressed. Gene functions were classified using KEGG and GO (101–103). The heat map was generated using Morpheus (https://software.broadinstitute.org/morpheus/).

### Quantitative reverse transcriptase PCR

Total RNA was extracted from cells using TRIzol Reagent (Invitrogen). cDNA was synthesized using either the iScript cDNA Synthesis Kit (Bio-Rad) with random hexamers or the RevertAid kit (Thermo Fisher Scientific). Real-time PCR (qPCR) was performed with iTaq Universal Supermix (Bio-Rad) on a CFX Connect Real-Time PCR Detection System (Bio-Rad). Primer sequences used are listed in Table 1. Relative mRNA levels were determined as described (104). To verify the efficiency and accuracy of amplification, standard curves were generated for each primer set on each PCR plate using serial dilutions of an appropriate experimental sample.

**TABLE 1:** Primers used for real-time PCR

Gene	Sequences^*a*^
*UBE2D2*	F: 5′-TGCCTGAGATTGCTCGGATCTACA R: 5′-ACTTCTGAGTCCATTCCCGAGCTA
*ACKR3*	F: 5′-ATCCTGCACTACATCCCTTTC R: 5′-CTTCATCAGCTCGTACCTGTAG
*BCL2*	F: 5′-GTGGATGACTGAGTACCTGAAC R: 5′-GAGACAGCCAGGAGAAATCAA
*COTL1*	F: 5′-TCCAAGTTTGCCCTCATCAC R: 5′-GAAATCTTCCTCCAGCTCCTTC
*ITGA4*	F: 5′-CTGTGGACCTCAATGCAGAT R: 5′-CACTTCCAACGAGGTTTGTTTC
*KITLG*	F: 5′-CTGCAGGAATCGTGTGACTAATA R: 5′-CTACCATCTCGCTTATCCAACAA
*LAMA1*	F: 5′-AGAGAATGGAGTGAGACAGGA R: 5′-CGATGCCTTGATGAGGATGT
*PDLIM1*	F: 5′-AGGAGAAACAGGAGTTGAATGAG R: 5′-ATCGACGCAGCCACTTTAG
*S100A4*	F: 5′-TCAAGCTCAACAAGTCAGAACTA R: 5′-CAGGAAGACACAGTACTCTTGG
*SDC1*	F: 5′-GGATGACTCTGACAACTTCTCC R: 5′-GAATAGCCGTCAGGAGCTG
*SDC4*	F: 5′-CGTTGAAGAGAGTGAGGATGTG R: 5′-TGAGCAGTAGGATCAGGAAGA
*SMAD7*	F: 5′-AACTGCAGACTGTCCAGATG R- 5′-CTCGTCTTCTCCTCCCAGTAT
*STAT3*	F: 5′-CACATGCCACTTTGGTGTTTC R: 5′-GCTTCTCAAGATACCTGCTCTG
*ITGβ1*	F; 5′-CAAAGGAACAGCAGAGAAGC R: 5′-ATTGAGTAAGACAGGTCCATAAGG

^*a*^ F, forward; R, reverse.

### Flow cytometry and cell size comparisons

Cells were adjusted to 5 × 10⁵ cells/mL and cultured for 24 h prior to analysis. For each labeling reaction, 5 × 10⁵ cells were collected by centrifugation at 800 × *g* and 4°C for 3 min. Cells were resuspended in 2 mL of cold PBS/0.2% BSA (FACS buffer), centrifuged, and then resuspended in 50 μL of cold FACS buffer, which was supplemented with 2 μg anti-ITGA-4 (eBioscience, Catalog #16-0499-85). Cell/antibody suspensions were chilled on ice for 1 h and then washed with 2 mL of FACS buffer. Cell pellets were resuspended in 50 μL of FACS buffer, which was supplemented with 0.25 μg of Allophycocyanin (APC)-conjugated goat anti-mouse Ig (Southern Biotech). Cell/antibody suspensions were chilled on ice for 30 min. Cells were then washed with 2 mL of FACS buffer and fixed with PBS/2% paraformaldehyde at 4°C for at least 30 min. Following fixation, cells were washed in 2 mL of FACS buffer, resuspended in 500 μL of FACS buffer, and analyzed using a Cytek Aurora flow cytometer (Cytek Biosciences). Data were analyzed using FlowLogic Software (version 8.7; Inivai Technologies). To analyze cell size, 1 × 10^6^ cells were collected, washed in PBS, and resuspended in 1 mL PBS. Cells were then treated with LIVE/DEAD Fixable Blue Dead Cell Stain (Invitrogen) as described by the manufacturer and fixed. Variations in cell size were determined by differences in forward scatter. Cell size was separately measured using a Scepter cell counter (Millipore) with a 40 or 60 µm sensor. The instrument’s mean measurements were used.

### VCAM-1 adhesion assays

Recombinant human VCAM-1-Fc chimera (BioLegend 553704) was reconstituted in PBS to 100 µg/mL. For assays, an aliquot of the stock solution was diluted to 2.6 µg/mL in 0.1M NaHCO3 (pH 8.4), and 100 µL/well of the dilution was added to 96-well plates, which were chilled overnight at 4°C. Wells were then washed with 0.1% BSA in RPMI 1640 medium (wash buffer), blocked with 0.5% BSA in RPMI 1640 medium (block buffer) for 1 h at 37°C/5% CO_2_, and rewashed with wash buffer. The VCAM-1-coated plates were stored on ice until needed. For control assays, wells were coated with a thin gel layer of Cultrex according to the manufacturer’s instructions. HTLV-1-infected SLB-1 cells stably expressing shGFP or shMYOF and Jurkat cells were cultured overnight and then resuspended in PBS at 1 × 10^6^ cells/mL. Cells were stained with 2.5 μM Calcein AM (Thermo Fisher Scientific) for 20 min at 37°C, protected from light. Cells were then centrifuged and resuspended in pre-warmed RPMI 1640 supplemented with 1.8 mM CaCl_2_ and MgCl_2_ at 1 × 10^6^ cells/mL and incubated for 10 m at 37°C. Cells (5 × 10^4^) were added to VCAM-1 or Cultrex-coated wells and incubated for 1 h at 37°C/5% CO_2_. Wells were washed three times with wash buffer, and cells were lysed with 100 μL 1× Passive Lysis Buffer (Promega). Fluorescence intensity was measured at 488/520 nm using a Varioskan LUX plate reader with SkanIt 7.1 software (ThermoFisher Scientific).

### F-actin staining

Wells of µ-slide 8-well chamber slides (ibidi 80807) were coated with poly-L-lysine (MilliporeSigma P1399) as described by the manufacturer. Cells were equalized at 5 × 10^5^ cells/mL and cultured overnight. Cells were then centrifuged, resuspended in unsupplemented RPMI 1640 medium to 2 × 10^6^ cells/mL, and added to the slide chambers (300 µL/chamber). Slides were incubated at 37°C/5% CO_2_ for 15 min. Supernatants were removed, and cells were fixed with cold PBS/4% paraformaldehyde on ice for 30 min. Cells were washed twice with PBS, permeabilized in PBS/0.1% Triton X-100 at room temperature for 15 min and then washed twice with PBS. Cells were stained with Alexa Fluor 488 Phalloidin (Invitrogen A12379) as described by the manufacturer and counterstained with 30 µg/mL of DAPI in PBS. Cells were overlaid with ibidi Mounting Medium and imaged with a LSM 900 confocal microscope (Zeiss) using a 40× objective. Within experiments, the same laser power and gain settings were used for each specimen. Three-dimensional images were done using Zen Lite (version 3.8; Zeiss). Relative F-actin levels were quantified from maximum intensity Z-projections using Fiji with the macro developed by Zonderland et al. (49).

### Infection assays

Infected cells were irradiated (77 Gy) using a MultiRad 350 X-Ray Irradiator, and then, 1 × 10^5^ of these effector cells were co-cultured with Jurkat pminLUC-viral CRE cells (2 × 10^5^) for 24 h (1 mL/well; 24-well plates). Cells were harvested, washed with PBS, and lysed with 100 μL of Passive Lysis Buffer (Promega). Lysates were normalized according to total protein, and luciferase activity was measured using the Luciferase Assay System (Promega) and a GloMax 20/20 Luminometer (Promega).

### Statistical analysis

Data were analyzed using JMP Pro 18. The Shapiro-Wilk test was used to test data for normality. Normally distributed data were analyzed using a standard, unpaired two-tailed *t*-test. Non-normally distributed data were analyzed using the Mann-Whitney U test. Specific tests used are indicated in the figure legends.

## Data Availability

All data supporting the findings of this study are available within the article and its supplemental material.

## SUPPLEMENTAL MATERIAL

Supplemental figures jvi.00264-26-s0001.pdf Fig. S1 to S4.

Table S1 jvi.00264-26-s0002.xlsx Genes downregulated in shMYOF cells compared to shGFP cells.

ASM does not own the copyrights to Supplemental Material that may be linked to, or accessed through, an article. The authors have granted ASM a non-exclusive, world-wide license to publish the Supplemental Material files. Please contact the corresponding author directly for reuse.
