# Supplementary material for: Myoferlin contributes to invasiveness of human T-cell leukemia virus type 1-infected T cells
Source: J Virol. 2026 Apr 21;100(5):e00264-26. doi: 10.1128/jvi.00264-26 (PMC13185549; doi:10.1128/jvi.00264-26)
Supplement: Supplemental figures — Fig. S1 to S4. [file jvi.00264-26-s0001.pdf]

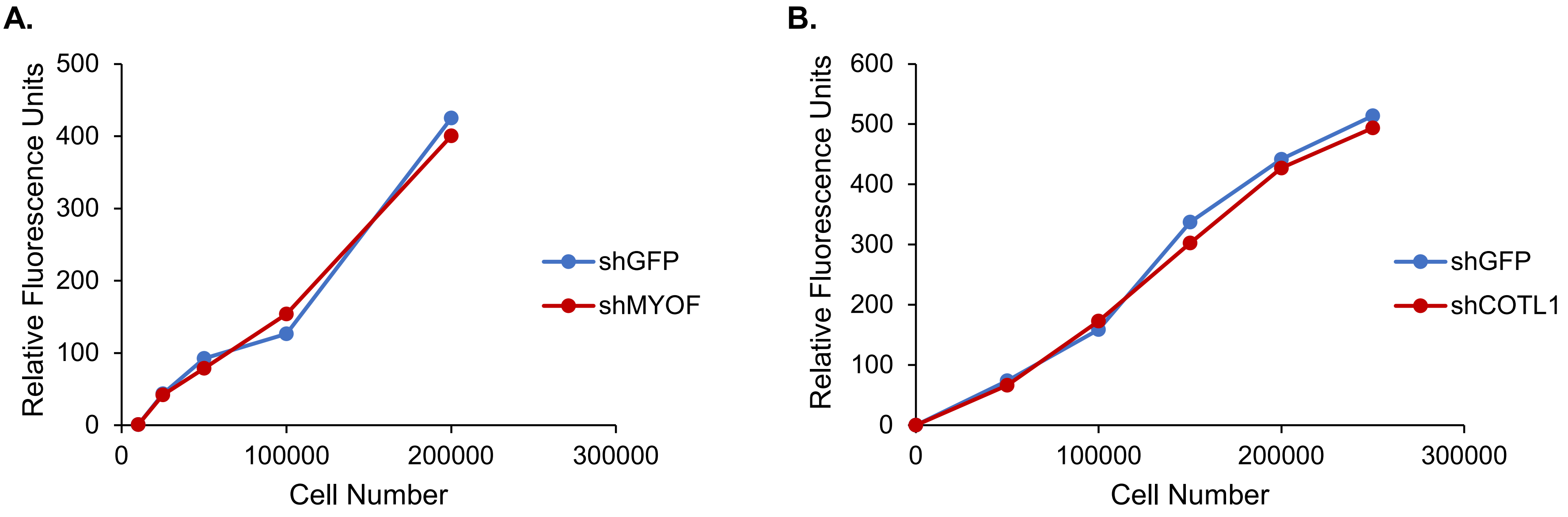

Figure S1: Different quantities of Calcein AM-labeled SLB-1 cells stably expressing an shRNA targeting GFP (negative control), **(A)** MYOF or **(B)** COTL1 mRNAs were lysed, and fluorescence was measured. Graphs show fluorescence values in function of cell number.

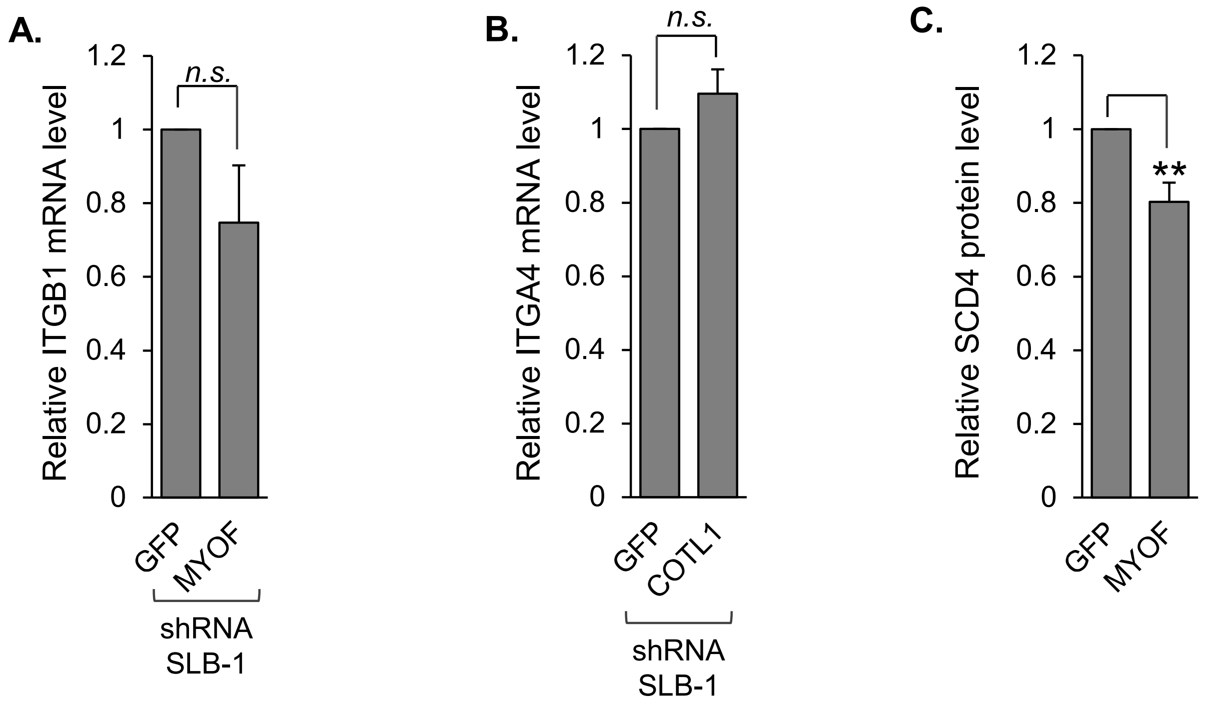

Figure S2: (A) Relative ITGB1 mRNA levels in SLB-1 shGFP or shMYOF stable cell lines. The graph shows qRT-PCR results averaged from three independent experiments with values normalized to that of shGFP (set to 1) for each replicate. **(B)** Relative ITGA4 mRNA levels in SLB-1 shGFP or shCOTL1 stable cell lines. The graph shows qRT-PCR results averaged from three independent experiments with values normalized to that of shGFP (set to 1) for each replicate. **(C)** The graph shows quantification of band intensities of SDC4 normalized to band intensities of β-actin averaged from three independent experiments for SLB-1 cells, and relative to shGFP (set to 1). Error bars show standard deviations; ** p<0.01 (t-test).

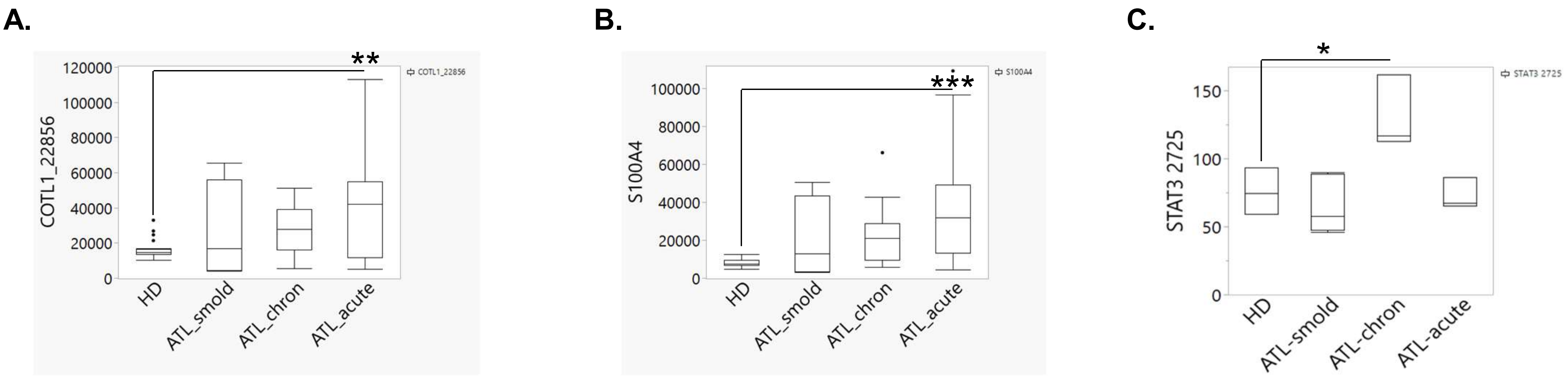

Figure S3: Tukey boxplots show relative **(A)** COTL1 and **(B)** S100A4 transcript levels from CD4$^+$ T-cells collected from healthy donors (HD) and patients with smoldering ATL, chronic ATL or acute ATL. Graphs were constructed using published microarray data for GEO accession number GSE33615 (40). **(C)** The Tukey boxplot shows relative STAT3 transcript levels. The graph was constructed using published microarray data for GEO accession number GSE55851 (77). Boxplot ends of each whisker are set to 1.5 times the interquartile range above the third quartile and below the first quartile; * p<0.05, ** *p*<0.01, *** *p*<0.001 (one-way ANOVA, Tukey HSD *post hoc* test).

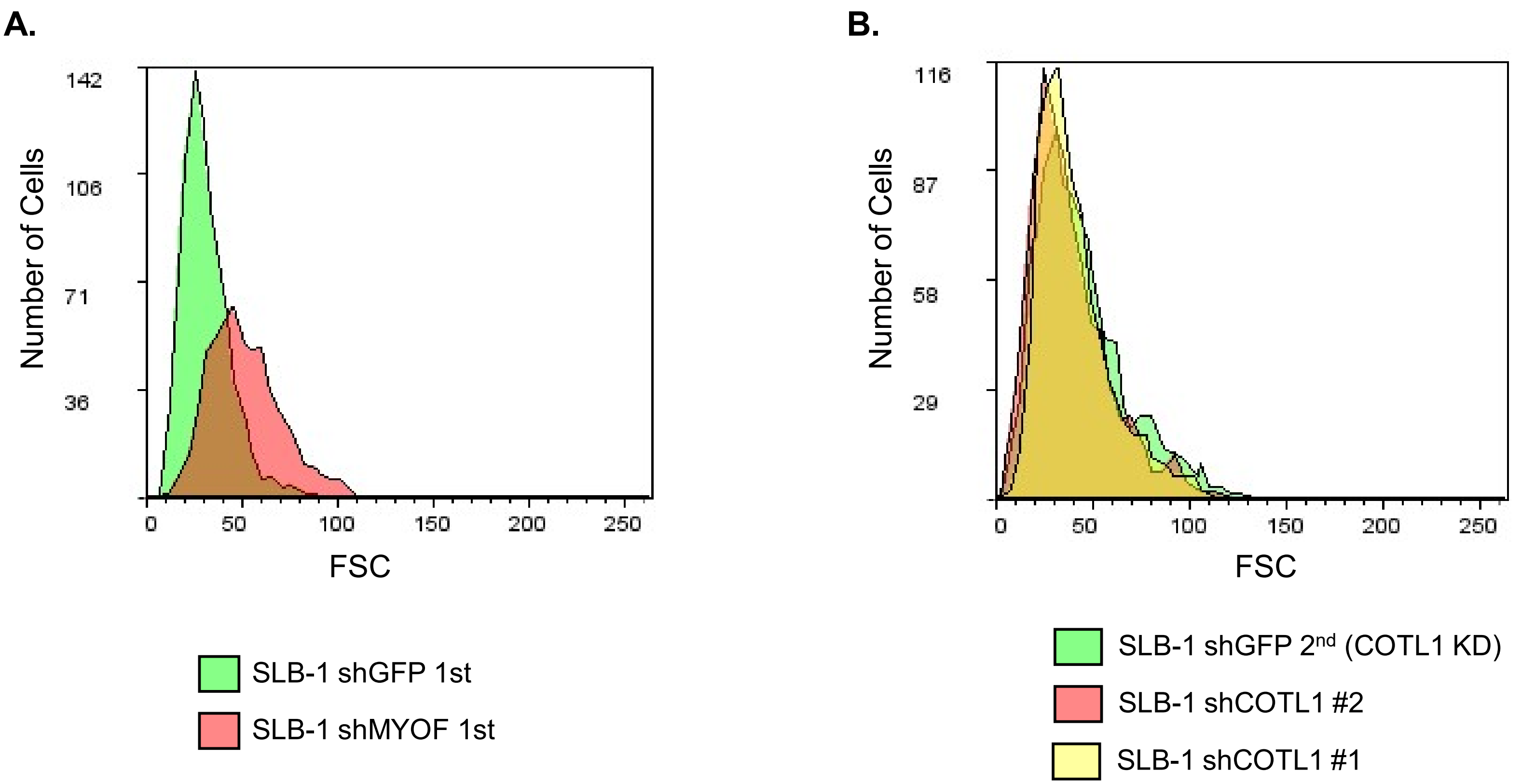

Figure S4: Depletion of MyoF but not COTL1 leads to an increase in cell size. SLB-1 cells stably expressing shRNA targeting **(A)** GFP (shGFP) or MYOF mRNA (shMYOF), or **(B)** GFP (shGFP) or COTL1 mRNA (shCOTL1) were treated LIVE/DEAD Fixable Blue Dead Cell Stain, fixed and analyzed by flow cytometry. The histograms show forward scatter of live cells.
